# Supplementary material for: Validation of a culturally sensitive, Swahili-translated instrument to assess suicide risk among adults living with HIV in Tanzania
Source: Glob Ment Health (Camb). 2023 Oct 5;10:e67. doi: 10.1017/gmh.2023.59 (PMC10643229; doi:10.1017/gmh.2023.59)
Supplement: Minja et al. supplementary material [file S2054425123000596sup001.docx]

**Supplementary Material**

**Table S1:** 3-Factor solution

|  |  | Factor 1 | Factor 2 | Factor 3 |
| --- | --- | --- | --- | --- |
| seasa2 | If you have serious thoughts of killing yourself in the future,  how confident are you that you will be able to keep  yourself from attempting suicide? | 0.94 |  |  |
| seasa4 | How certain are you that you could control future thoughts of suicide if you were experiencing physical or emotional pain? | 0.91 |  |  |
| seasa1 | How confident are you that you will not attempt suicide in the future? | 0.82 |  |  |
| seasa5 | How certain are you that you could control future suicidal thoughts if you lost an important relationship? | 0.84 |  |  |
| seasa6 | How certain are you that you could control future suicidal thoughts if you lost a job, could not find employment, or suffered a financial crisis? | 0.70 |  |  |
| seasa3 | If you have thoughts of killing yourself in the future, how confident are you that you will tell someone? | 0.44 |  |  |
| cssrs5 | Have you started to work out or worked out the details of how to kill yourself? Do you intend to carry out this plan? |  | 0.96 |  |
| cssrs6 | Have you ever done anything, started to do anything, or prepared to do anything to end your life? |  | 0.87 |  |
| cssrs4 | Have you had these thoughts and had some intention of acting on them? |  | 0.74 |  |
| cssrs3 | Have you been thinking about how you might do this? |  | 0.79 |  |
| brfl7 | I want to watch my children as they grow |  |  | 0.92 |
| brfl5 | I love and enjoy my family too much and could not leave them |  |  | 0.66 |
| brfl4 | The effect on my children could be harmful |  |  | 0.79 |
| brfl2 | My family depends upon me and needs me |  |  | 0.82 |
| brfl12 | I believe I can find purpose in life, a reason to live |  |  | 0.56 |
| brfl6 | My religious beliefs forbid it |  |  | 0.51 |
| brfl11 | I would not want people to think I did not have control over my life |  |  | 0.52 |
| brfl9 | I consider it morally wrong |  |  | 0.56 |

**Table S2:** 5-Factor solution

|  |  | Factor 1 | Factor 2 | Factor 3 | Factor 4 | Factor 5 |
| --- | --- | --- | --- | --- | --- | --- |
| seasa2 | If you have serious thoughts of killing yourself in the future,  how confident are you that you will be able to keep  yourself from attempting suicide? | 0.93 |  |  |  |  |
| seasa4 | How certain are you that you could control future thoughts of suicide if you were experiencing physical or emotional pain? | 0.89 |  |  |  |  |
| seasa1 | How confident are you that you will not attempt suicide in the future? | 0.87 |  |  |  |  |
| seasa5 | How certain are you that you could control future suicidal thoughts if you lost an important relationship? | 0.85 |  |  |  |  |
| seasa6 | How certain are you that you could control future suicidal thoughts if you lost a job, could not find employment, or suffered a financial crisis? | 0.68 |  |  |  |  |
| seasa3 | If you have thoughts of killing yourself in the future, how confident are you that you will tell someone? | 0.42 |  |  |  |  |
| cssrs5 | Have you started to work out or worked out the details of how to kill yourself? Do you intend to carry out this plan? |  | 0.94 |  |  |  |
| cssrs6 | Have you ever done anything, started to do anything, or prepared to do anything to end your life? |  | 0.84 |  |  |  |
| cssrs4 | Have you had these thoughts and had some intention of acting on them? |  | 0.83 |  |  |  |
| cssrs3 | Have you been thinking about how you might do this? |  | 0.81 |  |  |  |
| brfl7 | I want to watch my children as they grow |  |  | 0.68 |  | -0.48 |
| brfl5 | I love and enjoy my family too much and could not leave them |  |  | 0.68 |  |  |
| brfl4 | The effect on my children could be harmful |  |  | 0.85 |  |  |
| brfl2 | My family depends upon me and needs me |  |  | 0.80 |  |  |
| brfl12 | I believe I can find purpose in life, a reason to live |  |  | 0.35 |  |  |
| brfl6 | My religious beliefs forbid it |  |  |  |  |  |
| brfl8 | I am concerned about what others would think of me |  |  |  | 0.59 |  |
| brfl10 | I am afraid of the actual “act” of killing myself (the pain, blood, violence) |  |  |  | 0.68 |  |
| brfl11 | I would not want people to think I did not have control over my life |  |  |  | 0.64 |  |
| brfl9 | I consider it morally wrong |  |  |  | 0.76 |  |
| brfl1 | I am afraid of death |  |  |  |  | 0.56 |
| brfl3 | I do not want to die |  |  |  |  | 0.71 |

**Figure S1:** Items Endorsement

1. C-SSRS

| **Item** | **n(%)** |
| --- | --- |
| Have you wished you were dead or wished you could go to sleep and not wake up? |  |
| Yes | 80 (100) |
| No | 0 (0) |
| Have you had any actual thoughts of killing yourself? |  |
| Yes | 67 (83.8) |
| No | 13 (16.2) |
| Have you been thinking about how you might do this? |  |
| Yes | 48 (60.0) |
| No | 32 (40.0) |
| Have you had these thoughts and had some intention of acting on them? |  |
| Yes | 36 (45.0) |
| No | 44 (55.0) |
| Have you started to work out or worked out the details of how to kill yourself? Do you intend to carry out this plan? |  |
| Yes | 22 (27.5) |
| No | 58 (72.5) |
| Have you ever done anything, started to do anything, or prepared to do anything to end your life? |  |
| Yes | 22 (27.5) |
| No | 58 (72.5) |

1.
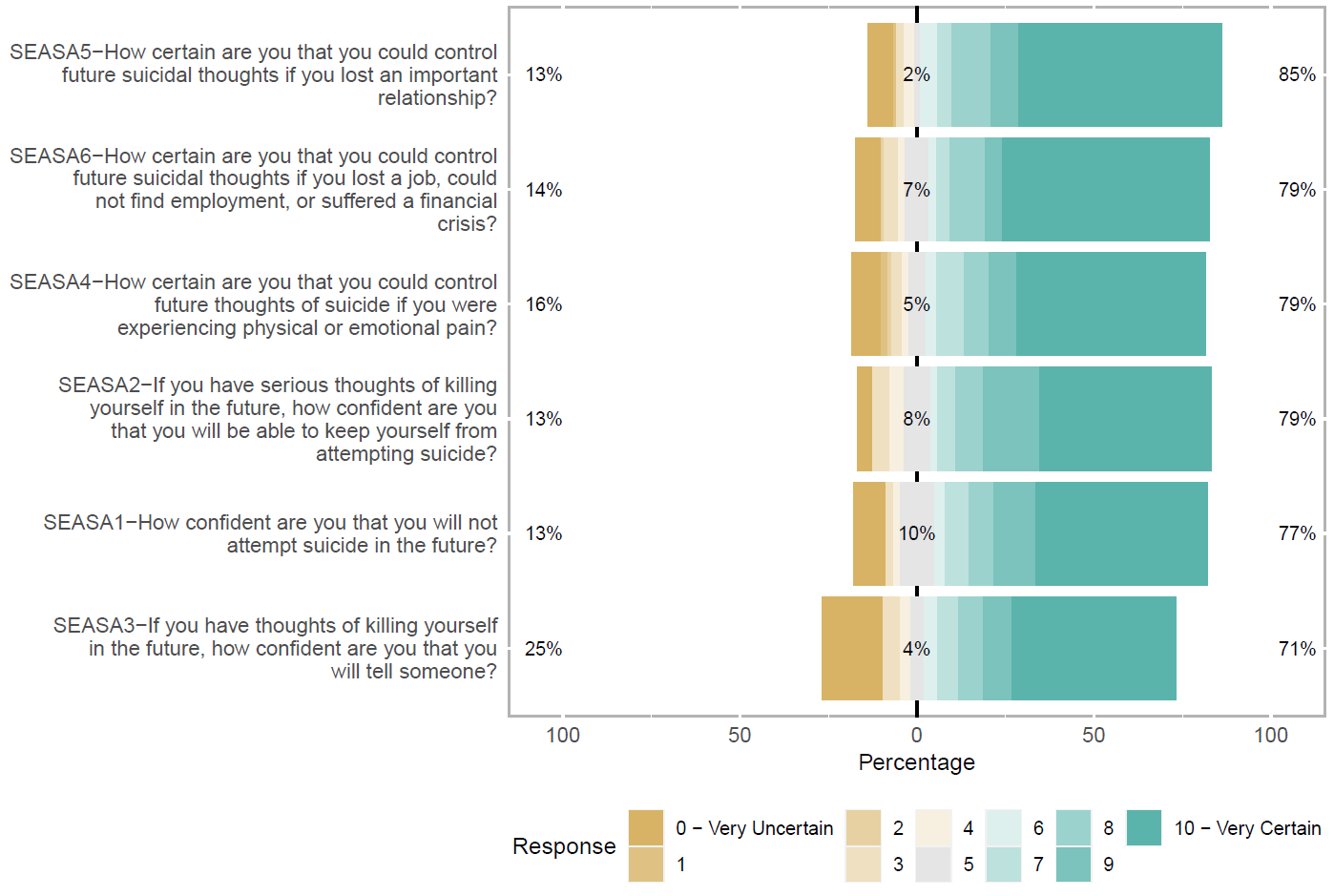
Self-Efficacy to Avoid Suicidal Action
2.
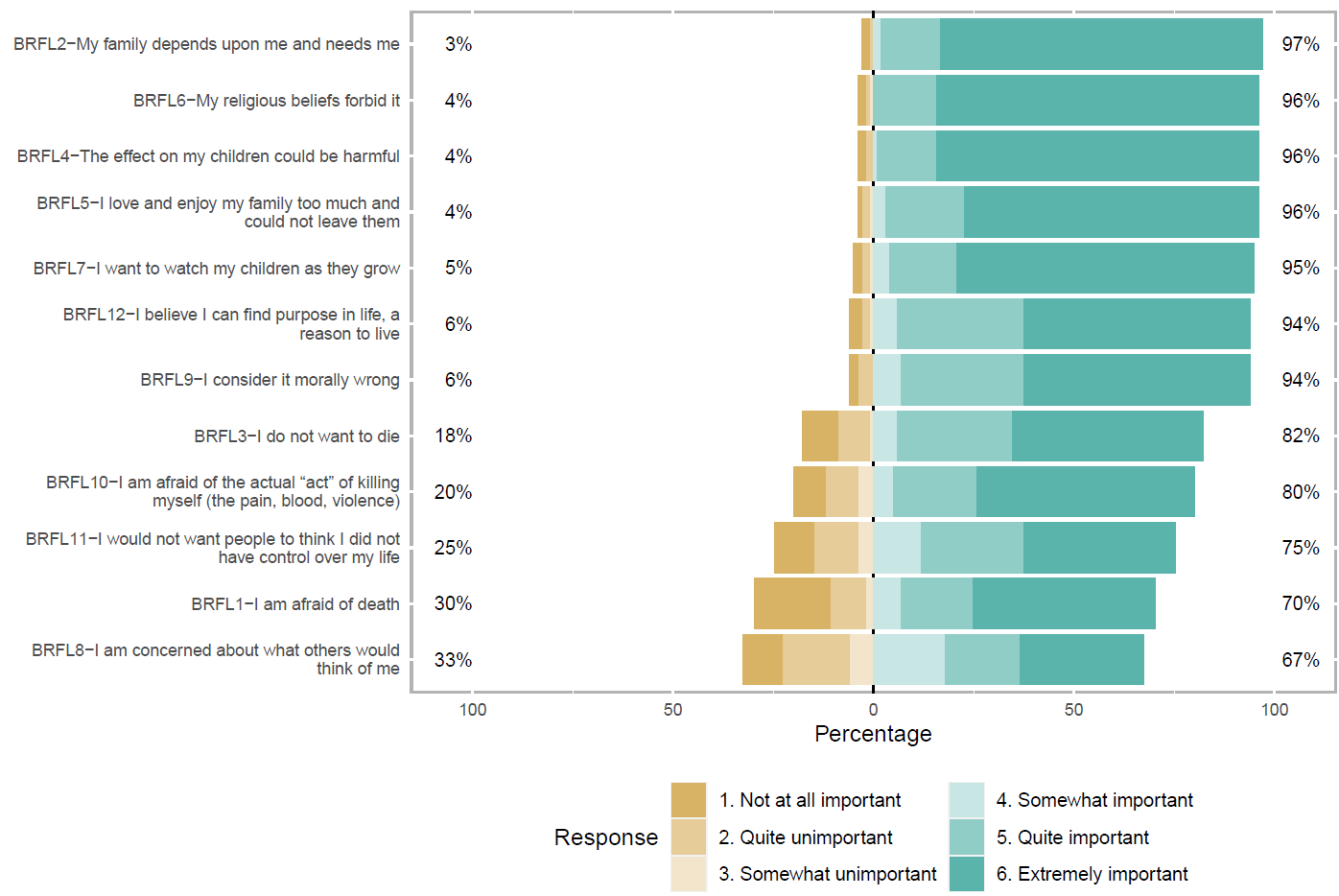
Brief Reasons for Living (BRFL)

**Figure S2:** Polychoric Matrix


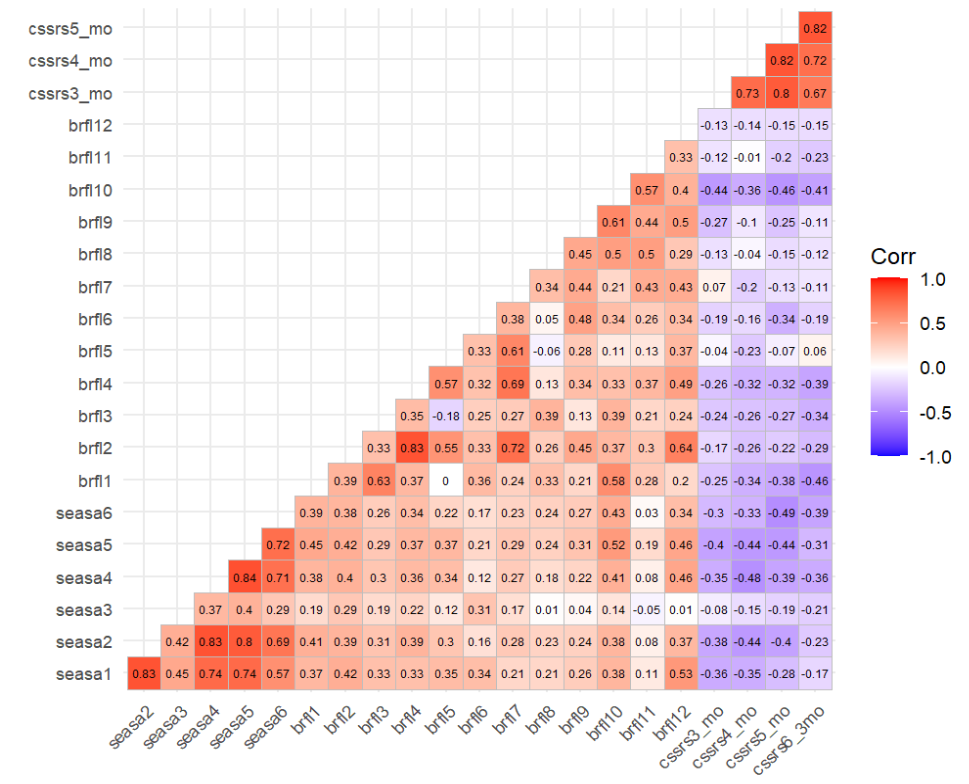


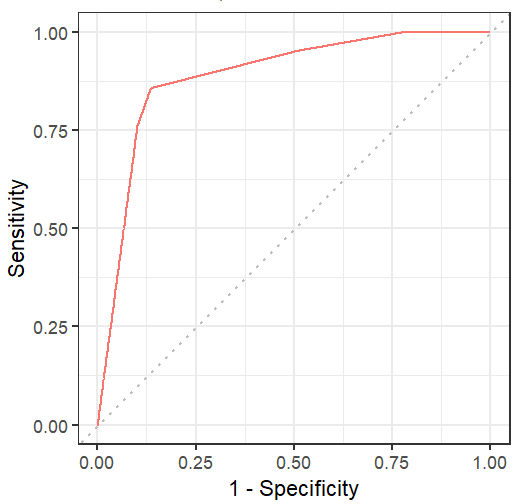

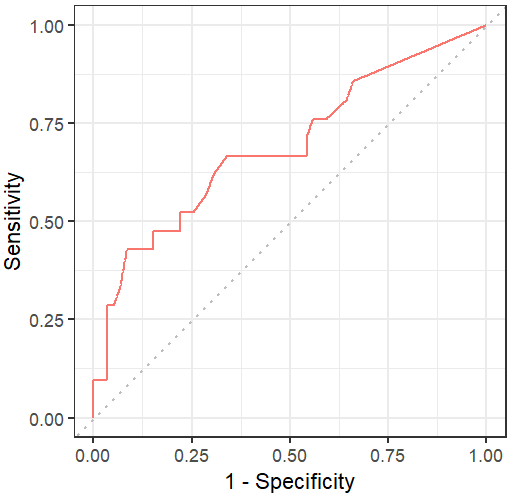
 **Figure S3:** ROC curves

Self-Efficacy

Intensity of suicidality


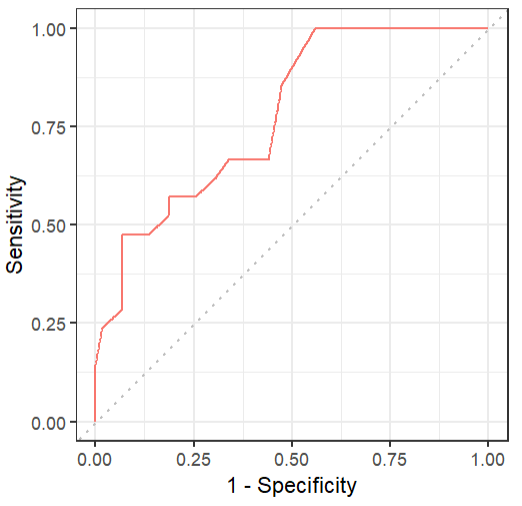

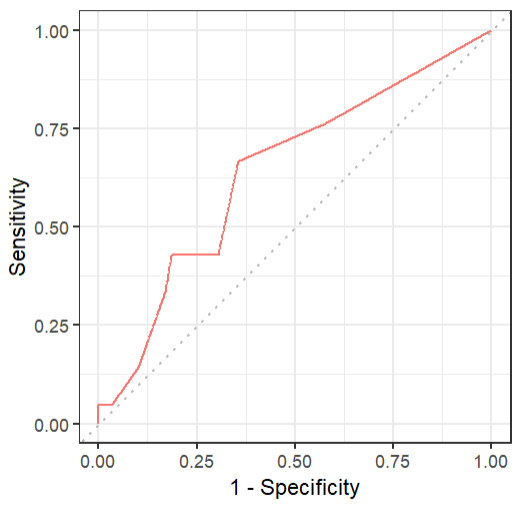


Fear and social concern

Family and spirituality
